# Species delimitation and phylogeny of a New Zealand plant species radiation

**DOI:** 10.1186/1471-2148-9-111

**Published:** 2009-05-20

**Authors:** Heidi M Meudt, Peter J Lockhart, David Bryant

**Affiliations:** 1 Allan Wilson Centre for Molecular Ecology and Evolution, Massey University, Private Bag 11222, Palmerston North 4442, New Zealand; 2 Department of Mathematics, University of Auckland, Private Bag 92019, Auckland 1142, New Zealand; 3 Museum of New Zealand Te Papa Tongarewa, PO Box 467, Wellington 6140, New Zealand

## Abstract

**Background:**

Delimiting species boundaries and reconstructing the evolutionary relationships of late Tertiary and Quaternary species radiations is difficult. One recent approach emphasizes the use of genome-wide molecular markers, such as amplified fragment length polymorphisms (AFLPs) and single nucleotide polymorphisms (SNPs), to identify distinct metapopulation lineages as taxonomic species. Here we investigate the properties of AFLP data, and the usefulness of tree-based and non-tree-based clustering methods to delimit species and reconstruct evolutionary relationships among high-elevation *Ourisia *species (Plantaginaceae) in the New Zealand archipelago.

**Results:**

New Zealand *Ourisia *are shown to comprise a geologically recent species radiation based on molecular dating analyses of ITS sequences (0.4–1.3 MY). Supernetwork analyses indicate that separate tree-based clustering analyses of four independent AFLP primer combinations and 193 individuals of *Ourisia *produced similar trees. When combined and analysed using tree building methods, 15 distinct metapopulations could be identified. These clusters corresponded very closely to species and subspecies identified on the basis of diagnostic morphological characters. In contrast, Structure and PCO-MC analyses of the same data identified a maximum of 12 and 8 metapopulations, respectively. All approaches resolved a large-leaved group and a small-leaved group, as well as a lineage of three alpine species within the small-leaved group. We were unable to further resolve relationships within these groups as corrected and uncorrected distances derived from AFLP profiles had limited tree-like properties.

**Conclusion:**

*Ourisia *radiated into a range of alpine and subalpine habitats in New Zealand during the Pleistocene, resulting in 13 morphologically and ecologically distinct species, including one reinstated from subspecies rank. Analyses of AFLP identified distinct metapopulations consistent with morphological characters allowing species boundaries to be delimited in *Ourisia*. Importantly, Structure analyses suggest some degree of admixture with most species, which may also explain why the AFLP data do not exhibit sufficient tree-like properties necessary for reconstructing some species relationships. We discuss this feature and highlight the importance of improving models for phylogenetic analyses of species radiations using AFLP and SNP data.

## Background

Volcanic island archipelagos, such as Hawai'i, Macaronesia, and Juan Fernández have long been the focus of studies investigating patterns and processes of species diversification (e.g., [1-8]). However, attention is increasingly being turned to the study of other small geologically older continental landmasses such as Madagascar, New Guinea, New Caledonia, and New Zealand [9-14], which are also spectacular biodiversity hotspots [15]. New Zealand hosts a unique flora, where over 85% of its native angiosperm species are endemic [16,17]. Endemism is greatest in the high-elevation habitats of its North and South Islands, where many genera have diversified following late Tertiary transoceanic dispersal of a single founder species to New Zealand [12,13,18-21].

These radiations have produced ecologically and morphologically distinct taxa, many of which have highly restricted distributions. Delimiting the boundaries of species within these radiations and evaluating their conservation status has been difficult [22,23]. This is because the criteria for recognizing species continue to be debated and because the phylogenetic relationships of species, as well as the genetic characteristics that distinguish them, are poorly understood [14,24,25].

The operational criteria used to define species boundaries, as well as the very nature of species, have been debated for more than 200 years (see [26-30] and references therein). Numerous species concepts have been proposed that emphasize different features considered important for delimiting species. This has led to different conclusions regarding species limits and the number of species in many groups [29,30]. Recently, a 'unified species concept' was advocated that emphasizes the common element found in many species concepts, which is that species are separately evolving lineages or metapopulations [29-32]. This unified concept also allows the use of diverse lines of evidence to test species boundaries (e.g., monophyly at one or multiple DNA loci, morphological diagnosability, ecological distinctiveness, etc. [28,29]) and is the species concept we adopt here.

New Zealand species of *Ourisia *(Plantaginaceae) are typical of many New Zealand alpine plant species radiations, as they are characterized by white zygomorphic to subrotate flowers, and polyploid species (hexaploids, 2*n *= 48) that occur in a diversity of habitats. These *Ourisia *species are found from sea level to 2300 m, most commonly in subalpine to alpine herbfields, tussock grasslands and scrub on moist to saturated, rocky, south-facing sites in the North, South and Stewart Islands. Some species are widespread, such as *Ourisia caespitosa *which is found on all three islands. Others are narrow endemics, such as *O. vulcanica*, found only on volcanic soils in the North Island, and *O. modesta*, a threatened and poorly known forest-dwelling species.

Analyses of morphological and DNA sequence data have been used to revise the taxonomy and test biogeographic hypotheses for 28 recognized species of *Ourisia *in the Southern Hemisphere [20,33,34]. Phylogenetic analyses of nuclear and chloroplast sequences suggest that the genus arose in South America and that it subsequently dispersed to Tasmania and New Zealand [20]. Because the New Zealand species form a monophyletic group, one event of transoceanic dispersal to New Zealand was hypothesized. In these published studies, which have been limited in the extent of their intraspecific sampling, little phylogenetic resolution was found within the New Zealand lineage [20,34]. For this reason, the New Zealand species have been delimited to date using traditional morphological methods only [33].

Here, we first use relaxed molecular clock analyses on nuclear ribosomal internal transcribed spacer region (ITS) sequences to determine the age of New Zealand *Ourisia *radiation. We then investigate the potential of amplified fragment length polymorphism (AFLP) analyses for delimiting New Zealand *Ourisia *species and reconstructing their evolutionary relationships. To do this, we use divergence plots and supernetworks to examine the tree-like properties of distances calculated from AFLP profiles and the extent of phylogenetic congruence in the AFLP data derived from four independent primer combinations. We then use both tree-based and non-tree-based clustering methods on the combined AFLP profiles to estimate the number of distinct metapopulations. We interpret our findings within the context of previous morphological studies [33] and discuss the potential and limitations of AFLP data analyses for delimiting species and inferring evolutionary relationships.

## Results

### Molecular dating the New Zealand *Ourisia *radiation with previously published ITS data

Relaxed clock analyses using BEAST v1.4.7, which assumed a mean substitution rate of 4.9 × 10^-9 ^substitutions/site for ITS sequences, suggest that the most recent common ancestor of the New Zealand *Ourisia *radiation is 0.8 million years old (95% HPD lower 0.4 mya, 95% HPD upper 1.3 mya), a finding consistent with a Pleistocene radiation of *Ourisia *species in New Zealand.

### Comparison of nuclear DNA sequence distances and AFLP distances

**Figure 1:**
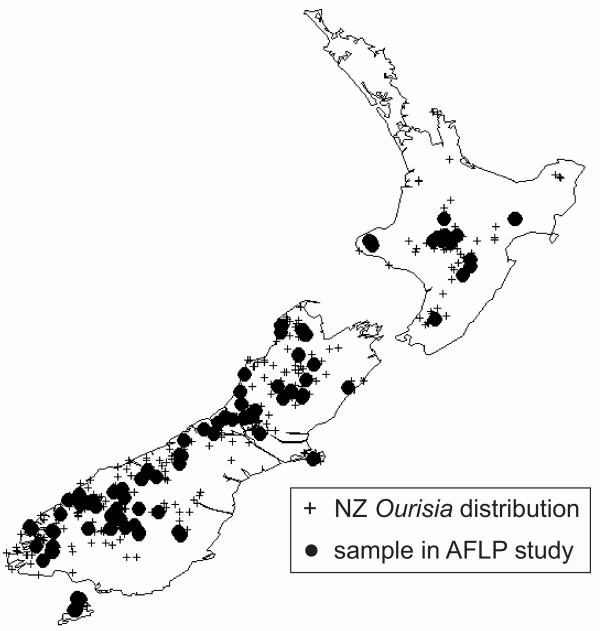
**Combined AFLP vs. nuclear DNA distances for Australasian species of *Ourisia***. Hamming, Dice, and Jaccard AFLP distances are plotted against Hamming distances for two nuclear DNA sequence markers. A. AFLP vs. ITS distances. B. AFLP vs. GBSSI distances.

AFLP profiles of New Zealand *Ourisia *did not diverge as a simple function of increasing DNA sequence divergence. A significant correlation was not observed between Hamming, Dice or Jaccard AFLP distances and Hamming distances obtained from nuclear DNA sequences (Fig. 1). Similar results were obtained irrespective of whether AFLP primer combinations were analyzed jointly or separately (data not shown). The formulae for the three different AFLP measures are very similar, and this was seen when the three different AFLP distance measures were plotted against one another (data not shown). Note that the normalization used in Jaccard and Dice distances gives them increased variance in comparison to Hamming distances, but the increased variance did not significantly lower bootstrap support for clades of clustered individuals, and support for relationships among clusters was generally low for both transformed and untransformed data.

### Individual datasets and supernetwork analyses

**Figure 2:**
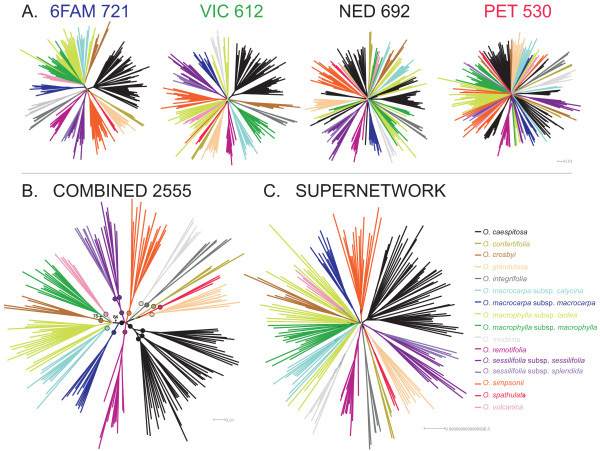
**Distance trees of the *Ourisia *combined AFLP data based on NJ bootstrap analyses using mean pairwise character distances (uncorrected, Hamming)**. A. Fifty percent majority rule bootstrap trees of the individual primer combination AFLP datasets. B. Fifty percent majority rule bootstrap tree of the combined AFLP dataset. Filled circles denote branches that have bootstrap support ≥92. C. Filtered supernetwork of four 50% majority rule bootstrap trees shown in A. Branch colour for all trees (A-C) denotes species and subspecies delimitation following Meudt [33] (as shown in the legend).

Tree-building analyses of AFLP data from 6FAM, VIC, NED and PET primers resulted in similar assignment of individuals into species clusters identified by diagnostic morphological characters (e.g., neighbour joining (NJ) trees in Fig. 2A; other data not shown). However, species relationships were poorly resolved, and even the large-leaved group (see below) was not supported by the majority of the analyses of individual primer combinations (although it was moderately to highly supported in some analyses of 6FAM and VIC) [see Additional file 1].

A Z-closure supernetwork was used to visualize the extent of incongruence between majority rule bootstrap trees built from NJ analysis of Hamming distances for AFLP profiles derived from 6FAM, VIC, NED and PET primers. This supernetwork was nearly star-like, with some (but not extensive) incongruence concerning relationships among species (Fig. 2C). Although many species relationships were not fully resolved, the large- and small-leaved groups were resolved (Fig. 2C). Inclusion in the analysis of 22 putative first and second generation hybrids greatly reduced the tree-like nature of the supernetwork (data not shown; Meudt et al. unpubl. data), suggesting that contemporary hybridization and gene flow is unlikely to be contributing to the lack of resolution observed with the reduced dataset. The finding of limited incongruence between the splits inferred from the individual data sets was used to justify combining the data.

### Phylogeny of New Zealand *Ourisia *based on combined AFLP data

**Figure 3:**
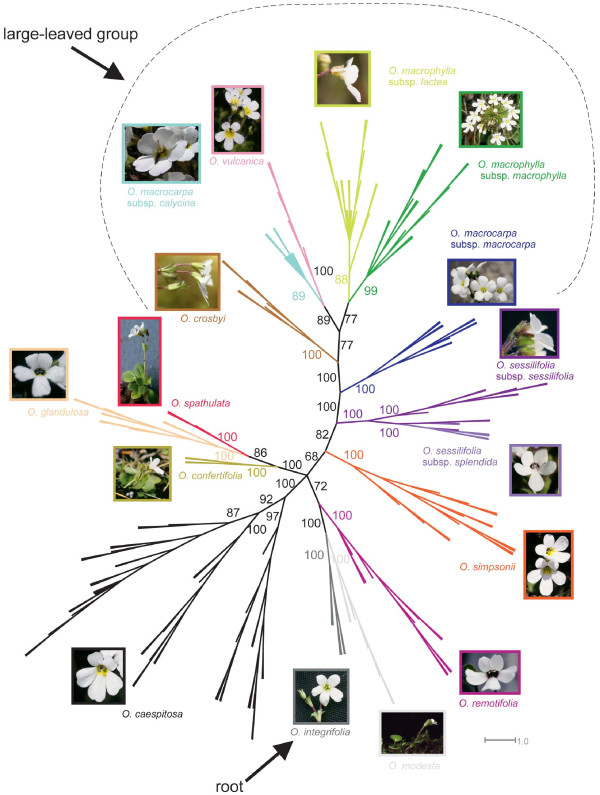
**Unrooted 50% majority rule tree from Bayesian analysis of the combined AFLP dataset for all New Zealand and Australian species of *Ourisia***. Posterior probability values ≥50 are shown near each branch. Colours follow Figure 3. *Ourisia integrifolia*, the sole Australian species, is the sister to all the New Zealand species [20] and thus the root of the tree. Location of the large-leaved group is also shown. All photos by HMM except *O. macrocarpa *subsp. *calycina *(P. Knightbridge) and *O. modesta *(C. Ogle).

Trees reconstructed with combined AFLP data from the New Zealand species of *Ourisia *using NJ (with different distance measures), maximum parsimony (MP), and Bayesian estimation generally had very similar topologies and support values (e.g., Figs. 2B and 3; Table 1). All trees contained a highly supported split separating two main groups – roughly characterized by leaf size, inflorescence type, and corolla tube characters – but otherwise had low phylogenetic resolution. Thus, a lineage comprising *O. crosbyi, O. macrocarpa, O. macrophylla*, and *O. vulcanica *– hereafter referred to as the large-leaved group – was highly supported (91–100 bootstrap percentage (BP)/100 posterior probability (PP); Table 1; Figs. 2B and 3) and (in rooted trees) [see Additional file 2] nested within a small-leaved group comprising the remaining New Zealand species (sometimes except *O. modesta*).

**Table 1:** Comparison of bootstrap values for monophyletic species and subspecies and certain species relationships for different tree-building analyses of the *Ourisia *combined AFLP dataset

	**MrBayes**	**PAUP**	**PAUP**	**SplitsTree**	**SplitsTree**	**TREECON**	**TREECON**
**SPECIES**	**restriction model**	**MP**	**NJ Hamming**	**NJ Dice**	**NJ Jaccard**	**NJ Nei Li**	**NJ Link**
*Ourisia caespitosa*	100	95.6	100	100	100	100	100
*O. confertifolia*	100	100	100	100	100	100	100
*O. crosbyi*	100	100	100	99	100	100	100
*O. glandulosa*	100	98.3	94	100	100	99	98
*O. macrocarpa*	-	-	-	-	-	-	-
*O. macrocarpa *subsp. *calycina*	89	81.8	95	97	90	98	93
*O. macrocarpa *subsp. *macrocarpa*	100	100	100	100	100	100	100
*O. macrophylla*	-	65.4	-	-	-	-	-
*O. macrophylla *subsp.*lactea*	88	-	-	-	-	-	-
*O. macrophylla *subsp. *macrophylla*	99	73.5	75	77	70	74	74
*O. modesta*	100	100	100	100	100	100	100
*O. remotifolia*	100	100	100	100	100	100	100
*O. sessilifolia*	100	100	100	100	100	100	100
*O. sessilifolia *subsp. *sessilifolia*	-	-	-	-	-	-	-
*O. sessilifolia *subsp. *splendida*	-	-	-	-	-	-	-
*O. simpsonii*	100	100	100	100	100	100	100
*O. spathulata*	100	100	100	100	100	100	100
*O. vulcanica*	100	95.1	93	94	96	94	97
*O. integrifolia *(Tasmania)	100	100	100	100	100	100	100
							
**SPECIES RELATIONSHIPS**							
large-leaved group	100	73.6	98	98	95	100	91
large leaved group except *O. macrocarpa *subsp. *macrocarpa*	100	-	64	61	54	60	48
large leaved group + *O. sessilifolia*	82	-	-	73	79	68	75
large leaved group + *O. sessilifolia + O. simpsonii*	68	-	-	-	-	-	-
*O. glandulosa *+ *O. spathulata*	86	63.9	96	99	100	99	100
*O. glandulosa + O. spathulata + O. confertifolia*	100	86.6	98	97	99	98	99
*O. glandulosa + O. spathulata + O. confertifolia + O. modesta + O. integrifolia*	-	-	-	73	58	-	-
*O. remotifolia + O. modesta + O. integrifolia*	72	-	-	-	-	-	-
*O. modesta + O. integrifolia*	100	70.9	59	65	59	71	56
*O. sessilifolia *1 (8 individuals of subsp. *sessilifolia*, southern South Island)	100	100	100	100	100	100	100
*O. sessilifolia *2 (5 individuals of subsp. *splendida *+ 4 individuals of subsp. *sessilifolia*, central South Island)	100	99.7	100	100	100	100	100
*O. caespitosa *1 (11 individuals, central North Island)	100	90.7	98	100	98	100	99
*O. caespitosa *2 (16 individuals, Otago)	97 (97 +218a)	83.3	98	98	97	93	96
*O. caespitosa *3 (23 individuals, southern South Island + North Island)	87 (-218a)	63.1	92	95	92	92	92
*O. caespitosa*1 + 3	92	61.4	68	66	58	-	67

Species relationships within each of these two main groups were not well resolved or supported, with the notable exception of one highly supported alpine lineage comprising *O. glandulosa *+ *O. spathulata *+ *O. confertifolia *(99 BP/100 PP) in the small-leaved group. There was also high support for grouping individuals by species for 12 of the 13 species morphologically-delimited species [33] (Table 2, Fig. 3), including the South Island alpine species *O. spathulata, O. glandulosa, O. confertifolia, O. simpsonii, O. remotifolia*, and *O. sessilifolia*, the North Island *O. vulcanica*, as well as *O. caespitosa, O. macrophylla, O. modesta, O. crosbyi*, and the Tasmanian species *O. integrifolia *(Figs. 2B and 3)*. O. macrocarpa *was not monophyletic. Its two subspecies were reciprocally monophyletic with high support values, but they were not each others' closest relative. The two subspecies of *O. macrophylla *were reciprocally monophyletic in some analyses (or poorly resolved within *O. macrophylla*), and *O. sessilifolia *subsp. *sessilifolia *was paraphyletic with individuals of subsp. *splendida *nested within it (Table 2, Fig. 3). Thus, not all geographically allopatric and morphologically distinct subspecies were fully resolved by the tree-building analyses. Interestingly, there was high support in tree-building analyses for some geographic lineages within *O. sessilifolia *(southern vs. central parts of the South Island) and *O. caespitosa *(central North Island, southern South Island + North Island, and Otago) that do not appear to correspond entirely to morphological patterns (Fig. 3) [see Additional file 2].

**Table 2:** Comparison of species boundaries for New Zealand and Australian *Ourisia *using different methods

**Morphology **[33]	**Tree-building analysis**	**Structure analysis**	**PCO-MC analysis**	**Species delimitation (this paper)**
*Ourisia caespitosa*	✓ (3 lineages)	✓ (2 clusters)	✓	no change, but further investigation of lineages within species is required
*O. confertifolia*	✓	✓	(not distinguished)^4^	no change
*O. crosbyi*	✓	(not distinguished)^1^	(not distinguished)^5^	no change
*O. glandulosa*	✓	(not distinguished)^3^	(not distinguished)^4^	no change
*O. macrocarpa*	NO	NO	NO	recognize *O. macrocarpa s.s. *(see below)
*O. macrocarpa *subsp. *calycina*	✓	✓	(not distinguished)^5^	recognize at species level, *O. calycina*
*O. macrocarpa *subsp. *macrocarpa*	✓	✓	✓	recognize at species level, *O. macrocarpa*
*O. macrophylla*	✓	(not distinguished)^1^	(not distinguished)^5^	no change
*O. macrophylla *subsp.*lactea*	✓	(not distinguished)^1^	(not distinguished)^5^	no change
*O. macrophylla *subsp. *macrophylla*	✓	(not distinguished)^1^	(not distinguished)^5^	no change
*O. modesta*	✓	✓	✓	no change
*O. remotifolia*	✓	✓	✓	no change
*O. sessilifolia*	✓	✓	✓	no change
*O. sessilifolia *subsp. *sessilifolia*	(poorly resolved)^2^	(not distinguished)^2^	(not distinguished)^2^	no change, but further investigation of lineages within species is required
*O. sessilifolia *subsp. *splendida*	(poorly resolved)^2^	(not distinguished)^2^	(not distinguished)^2^	no change, but further investigation of lineages within species is required
*O. simpsonii*	✓	✓	✓	no change
*O. spathulata*	✓	(not distinguished)^3^	(not distinguished)^4^	no change
*O. vulcanica*	✓	(not distinguished)^1^	(not distinguished)^5^	no change
*O. integrifolia *(Tasmania)	✓	✓	✓	no change

Comparison of species boundaries using morphology [33], tree-building analysis of combined AFLP data (Bayesian inference, Fig. 3), Structure analysis of combined AFLP data (*K *= 12, Fig. 5), and PCO-MC analysis of the combined AFLP data (Fig. 6), and the recommendation for species delimitation within *Ourisia *based on all analyses. Superscript numbers indicate the species and subspecies that are found in the same cluster in the tree-building, Structure, or PCO-MC analysis (and are therefore poorly resolved or not distinguishable): ^1^*Ourisia crosbyi, O. macrophylla *subsp. *lactea, O. macrophylla *subsp. *macrophylla*, and *O. vulcanica*; ^2^*O. sessilifolia *subsp. *sessilifolia *and subsp. *splendida*; ^3^*O. glandulosa *and *O. spathulata*; ^4^*O. confertifolia, O. glandulosa *and *O. spathulata*; ^5^*O. crosbyi, O. macrocarpa *subsp. *calycina, O. macrophylla *subsp. *lactea, O. macrophylla *subsp. *macrophylla*, and *O. vulcanica*.

### Genetic structure analyses of the combined AFLP data

**Figure 4:**
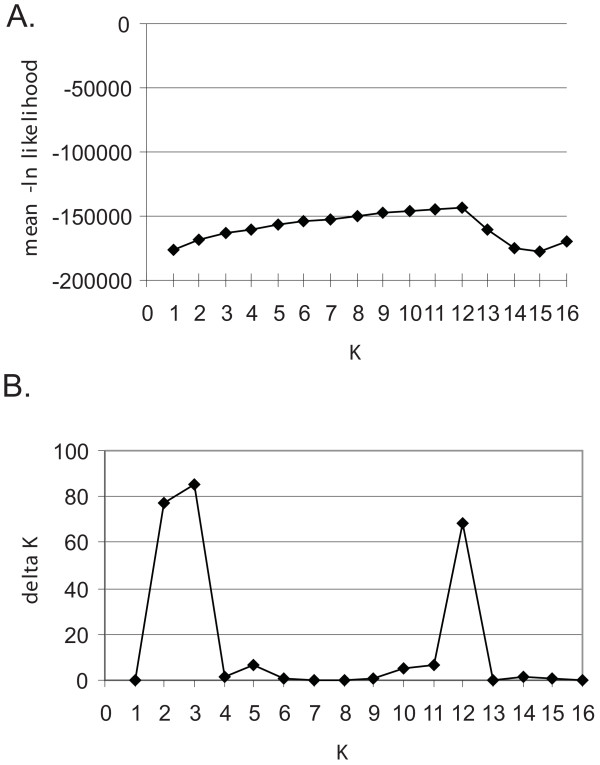
**Structure results from the *Ourisia *combined AFLP dataset**. A. *K *vs. mean -ln likelihood and B. *K *vs. Δ*K *for *K *= 1 to 16. Note the steady increase in -ln likelihood from *K *= 1 to *K *= 12 in A, and the multimodality of the Δ*K *values, with peaks at *K *= 2, *K *= 3, and *K *= 12.

**Figure 5:**
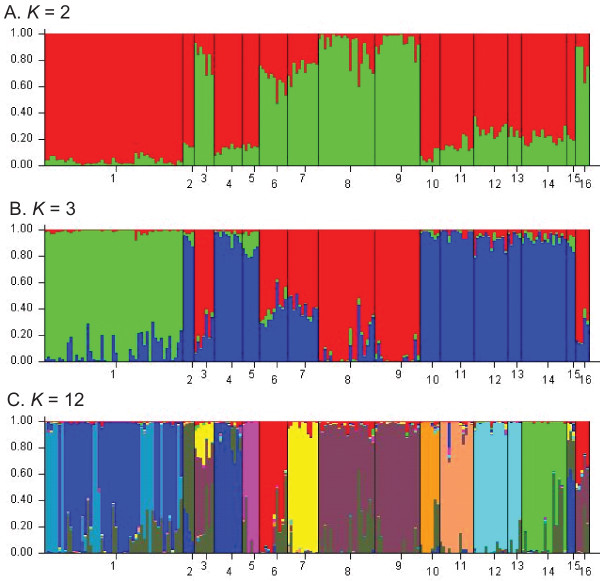
**Results of Structure clustering analyses for the *Ourisia *combined AFLP dataset for *K *= 2, *K *= 3, and *K *= 12**. *A priori *populations were defined as follows: 1, *Ourisia caespitosa*, 2, *O. confertifolia*, 3, *O. crosbyi*, 4, *O. glandulosa*, 5, *O. integrifolia*, 6, *O. macrocarpa *subsp. *calycina*, 7, *O. macrocarpa *subsp. *macrocarpa*, 8, *O. macrophylla *subsp. *lactea*, 9, *O. macrophylla *subsp. *macrophylla*, 10, *O. modesta*, 11, *O. remotifolia*, 12, *O. sessilifolia *subsp. *sessilifolia*, 13, *O. sessilifolia *subsp. *splendida*, 14, *O. simpsonii*, 15, *O. spathulata*, 16, *O. vulcanica*.

Bayesian clustering analyses with Structure identified several clusters that corresponded to morphologically-delimited species, subspecies and/or species groups for *K *= 2 to *K *= 12 (Table 2). For *K *= 13 to *K *= 16, analyses did not show increased resolution of species clusters (data not shown). Plots of the mean -ln likelihood vs. *K *(Fig. 4A) revealed that values increased from *K *= 1 to a maximum value at *K *= 12 (-143400.1), then decreased to a minimum value of *K *= 15 (-176974.7), and finally increased slightly at *K *= 16 (-169110.7). Plots of Δ*K *vs. *K *(Fig. 4B) showed the data had multiple peaks at *K *= 2 and *K *= 3 (Δ*K *= 77.565 and 85.331, respectively), and *K *= 12 (Δ*K *= 68.286). The Structure graphical output (from the run at each *K *with the highest likelihood) for these three values of *K *is shown in Fig. 5.

At *K *= 2 (Fig. 5A), the *Ourisia *individuals grouped into a large-leaved cluster comprising *O. crosbyi, O. macrocarpa, O. macrophylla*, and *O. vulcanica *and a small-leaved cluster of the remaining species, although the admixture levels for all individuals of the two subspecies of *O. macrocarpa *were high [see Additional File 3 and also below]. At *K *= 3 (Fig. 5B) individuals of *O. caespitosa *formed a separate third cluster. From *K *= 4 to *K *= 11, there was continued subclustering within both the small-leaved and large-leaved groups (data not shown). By *K *= 12 (Fig. 5C), nine of the clusters correspond to morphologically-delimited species or subspecies for *O. caespitosa *(comprising 2 clusters), *O. confertifolia, O. integrifolia, O. macrocarpa *subsp. *calycina, O. macrocarpa *subsp. *macrocarpa, O. modesta, O. remotifolia, O. sessilifolia*, and *O. simpsonii*, whereas the two remaining clusters comprised *O. crosbyi *+ *O. macrophylla *+ *O. vulcanica*, and *O. glandulosa *+ *O. spathulata *(Table 2). Taking all the Structure results into consideration [35,36], including Δ*K*, -ln likelihood, and individual assignment patterns, *K *= 12 appears to be the optimal number of clusters determined by Structure for all the values of *K *tested (*K *= 1 to 16).

Most pre-defined "populations" (species and subspecies) showed a high proportion of individuals assigned to one cluster only, generally from 73% to 95% [see Additional File 3]. Nevertheless, some "populations" had proportions much lower than this, and very few had proportions >95%. Thus, most species and subspecies contained levels of admixture much higher than the 5% threshold which might be attributed to stochastic noise. It is notable that in cases of high levels of admixture individuals were assigned to no more than 2 clusters. At *K *= 12, four species had levels of admixture >20% with one other species cluster, i.e. *O. confertifolia, O. crosbyi, O. spathulata*, and *O. vulcanica *(Fig. 5) [see Additional File 3]. *O. caespitosa *had similarly high levels of admixture (29%) to a second cluster, but this cluster largely comprised *O. caespitosa *individuals only (in other words, individuals of *O. caespitosa *were subdivided into two distinct and unique metapopulations).

**Figure 6:**
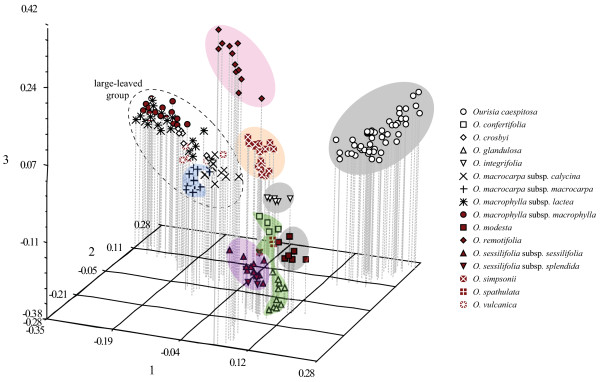
**Results of PCO-MC clustering analysis for the *Ourisia *combined AFLP dataset**. A three-dimensional plot of the principal coordinate analysis (PCO) from NTSYS using Jaccard distances is shown. *A priori *species and subspecies are shown in the legend. The eight clusters that were found to be significant in the PCO-MC analysis are shaded with colours that correspond to those in Fig. 2. The large-leaved group, which was not a found to be a significant cluster in the PCO-MC analysis, is encircled by a dashed line.

Most species were clearly separated into distinct clusters in the PCO analysis of the combined AFLP dataset (Table 2, Fig. 6). PCO-MC analysis showed that eight of these clusters (shaded in Fig. 6) were significant: *O. caespitosa, O. confertifolia *+ *O. glandulosa *+ *O. spathulata, O. integrifolia, O. macrocarpa *subsp. *macrocarpa, O. modesta, O. remotifolia, O. sessilifolia *(both subspecies), and *O. simpsonii*. By contrast, individuals of the five remaining species or subspecies of the large-leaved group – *O. crosbyi, O. macrocarpa *subsp. *calycina, O. macrophylla *subsp. *lactea, O. macrophylla *subsp. *macrophylla*, and *O. vulcanica *– could not be assigned to a significant cluster.

## Discussion

### AFLP analyses and species delimitation of New Zealand *Ourisia*

Our findings are consistent with earlier findings reporting the usefulness of AFLP analyses for distinguishing distinct metapopulations of individuals and delimiting species boundaries [37,32,41]. Of the 15 clusters identified by tree-building analyses of the combined AFLP dataset, 13 are highly supported lineages (99–100 PP; Fig. 3) and these correspond to 11 of the 13 Australasian *Ourisia *species and 2 of 6 subspecies recently delimited by morphological analyses [33]. Bayesian assignment (Structure) analyses (at the optimal *K *= 12 value; Table 2) identified nine species (clusters) also identified by the tree building analyses. The remaining three clusters identified by the Structure analyses (for *K *= 12) contained groups of species that were (in part) more fully resolved in the phylogenetic analyses. Thus, Structure produced congruent results to the tree-building analyses, but in contrast to expectations [42,43], provided less resolution in identifying distinct clusters. We speculate that this result might be explained by a poor fit between assumptions of the Structure model (Hardy-Weinberg equilibrium within populations and complete linkage equilibrium between loci) and our empirical data. The results from the PCO-MC analysis (Fig. 6) produced only eight significant clusters but were otherwise highly congruent to tree-building (Figs. 2B, 3), supernetwork (Fig. 2C) and STRUCTURE analyses (Fig. 5), including the difficulty in distinguishing among species in the large-leaved group.

Our AFLP tree topologies provide support for recent divergence of individuals belonging to the two subspecies of *O. macrophylla *and to the two subspecies of *O. sessilifolia*, respectively (Table 1, Fig. 3). This is a finding consistent with the taxonomic recognition of subspecies in these groups [44]. Subspecies status is appropriate here based on geographic and morphological distinctiveness [33]. For example, *O. macrophylla *subsp. *lactea *is a north-central South Island subspecies with glandular trichomes on the floral bracts and calyces, whereas subsp. *macrophylla *is subendemic to the North Island with eglandular trichomes on the floral bracts and calyces [33]. *O. sessilifolia *subsp. *splendida *is found in central South Island mountains and has only one line of hairs inside the corolla tube, whereas subsp. *sessilifolia *is disjunct in northern and southern parts of the South Island and has three lines of hairs inside the corolla tube). Nevertheless, obtaining samples of *O. sessilifolia *from the northern part of the South Island for inclusion in future genetic studies is key to fully understanding the overlapping patterns of morphological and genetic data reported here.

Interestingly, AFLP analyses suggest three distinct lineages within *O. caespitosa *that correspond to the following geographic areas: 1) central North Island, 2) southern South Island + North Island, and 3) Otago. There is also support for the sister relationship of the first two groups. Thus, the AFLP analyses provisionally support recognition of subspecific entities within *O. caespitosa*, including an Otago variety of *O. caespitosa *which has been recognized in the past [45,46] but not in the most recent treatment due to an apparent lack of morphological differences [33]. These intraspecific lineages require further genetic and morphological investigation across their distributional ranges to determine whether subspecific status is warranted.

In contrast to the above findings, we found no bootstrap support in tree-building analyses for a close relationship between the two recognised subspecies of *O. macrocarpa*. These AFLP data, together with DNA sequence data [20](Meudt et al. unpubl. data), suggest that the two subspecies comprise separate lineages that are not each others' closest relative. Further, the extent of divergence between these two allopatric lineages as indicated by the molecular data, coupled with the morphological differences that separate them – petiole and peduncle vestiture, leaf shape, leaf bases, calyx margins, and calyx symmetry [33] – comprise multiple lines of evidence that these lineages should be recognized as distinct species rather than subspecies, i.e. *O. macrocarpa *Hook.f. and *O. calycina *Colenso. If this interpretation is correct, it indicates that the morphological characters traditionally used to unite *O. macrocarpa *and *O. calycina *under one species, including large leaf lamina, fruits and flowers, oblanceolate to narrowly ovate calyx lobes, largely glabrous leaf lamina, and a lack of glandular hairs anywhere on the plant, may not be not good characters for species delimitation in the large-leaved group to which these species belong.

### AFLP analyses and evolutionary relationships among New Zealand *Ourisia*

Analyses of combined AFLP data identify a large- and small-leaved group, and distinguish a lineage of three alpine species within the small-leaved group. These inferences of evolutionary relationship are also supported by morphological and geographic data. Thus, the four species in the large-leaved group generally have the largest habits and biggest leaves (relative to the small-leaved group), flowers that are always or nearly always in whorls in each inflorescence node, and corolla tubes that are yellow and hairy inside [33]. Species in this lineage are generally found in forested to subalpine sites on the North, South and Stewart Islands of New Zealand. The large-leaved group contains *O. macrophylla *subsp. *macrophylla *and *O. vulcanica*, the only subspecies and species that are (sub)endemic to the North Island, which is known to have low floristic diversity and endemicity relative to the South Island (see discussion in [20]).

In contrast, the species in the small-leaved group have smaller habits and leaves, with flowers always or nearly always in pairs or solitary in each inflorescence node, and corolla tubes that are yellow and glabrous or purple and hairy inside [33]. Species in the small-leaved group (with the exception of *Ourisia modesta*) are largely high elevation, subalpine to alpine species on the South and Stewart Islands only. Within the small-leaved group, *O. glandulosa, O. spathulata *and *O. confertifolia *– which are highly supported as a monophyletic group in most AFLP analyses – have yellow and glabrous corolla tubes and are all southern South Island endemics. Based on morphology, the northern South Island endemic *O. simpsonii *could be included in this latter alpine group but there is no support for this in the AFLP analyses. *Ourisia modesta*, which is morphologically and ecologically distinct from all other New Zealand species [33], sometimes belongs to the small-leaved group, but in other analyses either pairs with the Tasmanian outgroup species *O. integrifolia *or is sister to all other species of New Zealand *Ourisia *(Table 1; Figs. 2 and 3).

### Limitations of AFLP analyses and inferences of phylogeny

Our analyses did not resolve a bifurcating phylogeny for the New Zealand species of *Ourisia*. The divergence plots for nuclear DNA sequences and AFLP profiles indicate that the AFLP-derived distances have limited tree-like properties. These plots show that the AFLP Hamming distances do not increase significantly with increasing sequence divergence (Fig. 1). This is in contrast to observations reported for other plant groups [47], and this finding contributes to the star-like features of the supernetwork and phylogenetic trees. The lack of resolution is not explained by an absence of signal in the AFLP data. Of the 2555 characters in the final combined AFLP dataset, 2365 (92.6%) are parsimony informative. Given these features of the data, we predict that reconstructed phylogenetic trees for New Zealand *Ourisia *are unlikely to become more tree-like (resolved as bifurcating) through increasing the number of AFLP characters. A previous study using nDNA (ITS, ETS) and cpDNA (*rps16 *and *matK *3' introns) sequencing markers did not resolve a bifurcating phylogeny of New Zealand *Ourisia *but had limited sampling (26 individuals; [20]). Two additional and more variable markers (nDNA GBSSI and cpDNA *trnC-D *intergenic spacer region) are being analyzed for a large number of individuals to further resolve the phylogeny and to compare to the AFLP results reported here (Meudt et al. unpubl. data).

Although Structure did not perform as effectively as tree-building in identifying clusters, Structure analyses do provide insight into the basis of the partially-resolved phylogenetic trees. In the case of a recent and rapid plant species radiation, it could be argued that the AFLP data have simply not evolved fast enough to accumulate substitutions and support species relationships. However, perhaps relevant to interpretation is the observation that Structure analyses indicate considerable admixture for many delimited metapopulations [see Additional File 3]. Stochastic noise in the AFLP data does not explain this result since the admixture is often >5% and is always between relatively few species. A possible explanation is hybridization and/or incomplete lineage sorting of ancestral polymorphisms among diverging species. If hybridisation is contributing to this signal we suggest that it is more likely to be ancient events of hybridisation between early diverging *Ourisia *lineages rather than contemporary gene flow. This follows from the observation that including putative recent hybrids with intermediate morphologies into the supernetwork analysis greatly increases the extent of incongruence in the network (Meudt et al. unpubl.). Thus, the inclusion of recent hybrids produces a different signature in the data analysis. Further, the species most implicated in recent hybridization events (*O. caespitosa*) does not show high levels of admixture, and the supernetwork shows very low levels of incongruence. The effect of ancient gene flow among early diverging lineages will be to make distance measures more similar than expected, and phylogenetic trees less tree-like. This speculation leads us to be cautious in interpreting the shape of the supernetwork and Bayesian tree topologies with respect to the pattern of species diversification. We believe their star-like features of the AFLP supernetwork and Bayesian tree reflect processes of diversification rather than the pattern of species radiation, an hypothesis that is being further investigated with nDNA and cpDNA sequencing markers (Meudt et al. unpubl. data).

## Conclusion

Phylogenetic, PCO-MC and Bayesian assignment (Structure) analyses of AFLP data for New Zealand *Ourisia *identified species and subspecies boundaries inferred previously from morphological analyses. In one case a change in rank from subspecies to species was proposed. In contrast to what has been suggested elsewhere, tree building analyses were found to be more efficient and robust in cluster identification than the Structure analysis [42]. Importantly, the results from the Structure analysis were informative for interpreting phylogenetic topology, and suggested that ancient gene flow among metapopulations is responsible for reducing the tree-like properties of the AFLP distance measures. If so, our observations may also explain some of the difficulties others have had in attempting to reconstruct the evolutionary history of species radiations using AFLP (e.g., [48-51]). Our findings highlight the need for developing analytical methodologies that explicitly take into account the evolutionary dynamics of rapid radiations. Such tools are needed for analyses of AFLP data as well as the rapidly growing collections of SNP data being generated by next generation sequencing technologies.

## Methods

### Generation and scoring of AFLP data

**Figure 7:**
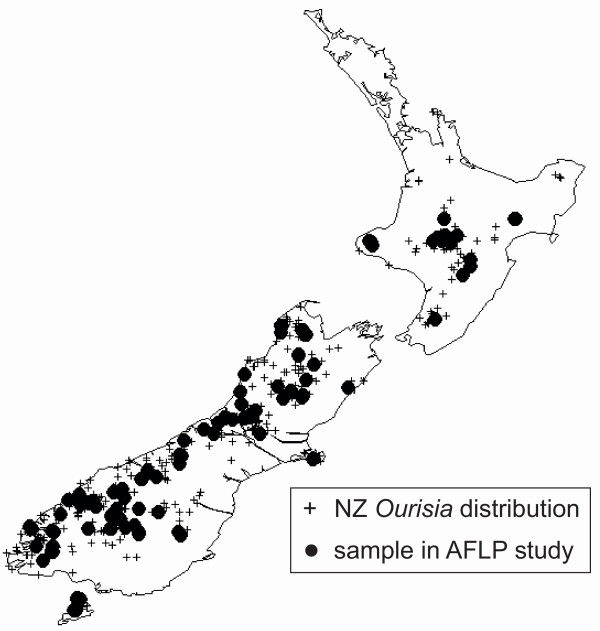
**Distribution of New Zealand species of *Ourisia***. Crosses represent distribution of all species in New Zealand; filled circles represent data sampled for AFLP.

Voucher specimens and leaf material dried on silica gel of 1–5 individuals from multiple populations were collected from throughout the entire geographic range of New Zealand *Ourisia *on all three main islands (Fig. 7) [see Additional file 4]. A total of 215 individuals were chosen for AFLP analyses, including six individuals from three populations of *Ourisia integrifolia*, the Tasmanian sister species of New Zealand *Ourisia *[20] which was used as an outgroup. DNA extractions were performed using DNeasy mini kits following the manufacturer's instructions, and only DNAs of high quality and high concentration (as checked on agarose gels) were used.

AFLP was performed following Meudt & Bayly [40] and also . Briefly, total DNAs were digested using EcoRI (Roche) and MseI (NEB) restriction enzymes for 37°C for 2 h (followed by 70°C for 15 m to denature the enzymes). Eco- and Mse-linkers were then ligated to the resulting DNA fragments by incubating with T4 DNA ligase (Roche) at 37°C for 3 h. Pre-selective PCR amplification was performed using primers Eco+A (0.5 μM) and 1.0 μL Mse+C (0.5 μM) and a stringent PCR program. Selective PCR amplification was performed using a fluorescently-labelled Eco+ANN primer (0.5 μM) and Mse+CNN primer (0.5 μM) and a step-down PCR program. Four different fluorescently-labelled Eco primers were used and trialled with all potential combinations of eight different Mse+CNN primers. The following primer combinations were chosen based on a screen involving 6 individuals: 6FAM-Eco+ACT/Mse+CCC, VIC-Eco+AGC/Mse+CGG, NED-Eco+ATA/Mse+CTG, and PET-Eco+AAG/Mse+CAG (hereafter, 6FAM, VIC, NED, and PET). All primers were from Sigma except VIC-, NED- and PET-labelled primers (Applied Biosystems). For each individual, selective amplifications of each of the four dyes were mixed together in equal amounts, and 1 μL of each sample was run on an Applied Biosystems Genetic Analyzer 3730 at the Allan Wilson Centre Genome Service. To ascertain reproducibility, 29 replicate AFLP profiles were generated for 22 individuals (ca. 10% of the dataset), either from independent DNA extractions of the same leaf material (9 individuals), or from independent restriction digests of the same DNA extraction (13 individuals, 20 replicate runs).

Automated scoring was performed on the resulting electronic profiles using GeneMapper v3.7 (Applied Biosystems) with scoring parameters that were optimized for a 30-individual representative subset of the entire dataset (including 6 replicate pairs) in [52]. The optimal scoring parameters were found to be bin width 0.5 bp, minimum fragment length scored 100 bp, and peak height threshold 50 rfu. Error rates and assignment of six of the 29 replicate AFLP profiles were thoroughly discussed in [52]. Therefore we only briefly note here the results of a NJ bootstrap analysis with 1000 fast bootstrap replicates of the data matrix (244 profiles, 2691 characters; data not shown). In this analysis, 80% of replicate profiles, including all but one of the replicate DNA extractions and most replicate digests, were correctly assigned with high bootstrap percentages as sister to their replicate pairs (21 replicates from 16 individuals, 87–100 BP) or in the same clade as their replicate pair plus one other individual from the same population (2 replicates from 2 individuals). With few exceptions, the Hamming distances of these replicate pairs were 0 to 0.08 (compared to 0 to 0.21405 for the entire dataset). By contrast, 20% of replicate profiles (6 replicates from 6 individuals) were either sister to individuals from other geographically proximate populations of the same species (67–94 BS) or unresolved. The Hamming distances of these 6 replicate pairs were generally from 0.10 to 0.16, and in all cases except one these were replicate runs based on the same DNA extraction. Upon visual inspection of the profiles, most variation between each replicate pair appeared to be due to differences in peak height (and shape) that result in scoring errors during the automated scoring process ([52]; pers. obs.).

Following scoring optimization and replicate checking, all 29 replicate profiles were removed; in addition, 22 putative natural hybrid individuals identified based on morphological characters were also removed and will be discussed in detail in a separate publication (Meudt et al. unpubl. data). This resulted in a final dataset of 193 individuals from multiple populations representing the ecological and morphological diversity of New Zealand *Ourisia *plus the Tasmanian outgroup. The combined binary matrix of 2555 characters (721, 6FAM; 692, VIC; 612, NED; 530, PET) was then exported and converted into a NEXUS file using GenotyperRearranger (W. Allen, unpubl., available at ).

### Comparison of AFLP distances and nuclear sequence data distances

Koopman [47] reviewed the potential of AFLP data for reconstructing the evolutionary histories of plants. He reported that the topologies of AFLP trees were largely congruent with ITS trees in several studies, and that AFLP was able to resolve with high bootstrap support relationships of individuals that differed by ca. 10–30 ITS nucleotide differences. Koopman [47] noted that when ITS sequence divergence was neither too small nor too large (i.e., divergence is approximately 3–5%) AFLP profiles appear to provide useful phylogenetic information. Nevertheless, the relationship between rates of divergence in AFLP and nuclear DNA sequence data – an important issue that has bearing on whether AFLP is appropriate for tree-building analyses – has not been critically examined. We investigated the relationship of AFLP-derived distances (Hamming and Jaccard and Dice transformed distances [53-55]) with ITS and GBSSI sequence divergences for New Zealand *Ourisia*. Hamming distances were calculated for ITS sequences representing 25 individuals obtained in an earlier study [20] and for GBSSI (part of *waxy*) sequences representing 65 individuals (Meudt et al. unpubl. data). Divergence plots were then obtained by comparing these distances with Hamming, Jaccard and Dice distances calculated from AFLP profiles. This was done for 25 individuals collected from the same populations/locality as the individuals sampled for ITS and from the same 65 individuals sequenced for GBSSI. Distances were obtained using SplitsTree 4.10 [56] and compared using a script written in R (S. Joly, unpubl.).

### Molecular dating analyses

To verify that New Zealand *Ourisia *comprise a recent, rapid radiation, and to provide a temporal context for interpreting genetic variation within the group, a relaxed molecular clock approach [57] was used to re-analyze previously published ITS data [20]. This analysis assumed a mean substitution rate for ITS sequences for ten herbaceous plant groups reported by [58] of 4.69 × 10^-9 ^substitutions/site (prior: normal distribution, standard deviation = 1.82 × 10^-9^), the optimal GTR + gamma (4 rate categories) substitution model, tree prior: Yule process, tree prior: tmrcaNZgroup, using a dataset with 42 individuals of *Ourisia *from South America, Australia, and New Zealand and the software BEAST v1.4.7. [57]. The chain length was 10,000,000 and convergence was monitored in independent runs using Tracer v1.4.1 .

### Tree-building analyses and assessment of data partition homogeneity

To address questions of species delimitation and relationships, AFLP data generated from multiple primer combinations are generally combined into one large matrix and analyzed together to produce a phylogenetic tree. This is often done without assessing the homogeneity of the data partitions (e.g., [41,51,53]). Although this issue has been extensively discussed in the literature when analysing sequences from independent gene loci, the issue is not often considered for individual AFLP characters (see discussion in [52]) or sets of characters (such as individual primer combinations). One advantage of analyzing the partitions separately is that it provides an opportunity to check for bias in phylogenetic estimates arising from any biological and/or technical reasons related to the AFLP protocol.

Phylogenetic trees were reconstructed for all individual primer datasets using maximum parsimony (MP), neighbour joining (NJ), and Bayesian inference methods. NJ trees were built using Hamming, Jaccard and Dice distances with SplitsTree 4.10 [56] and TREECON v1.3b [59]. MP trees were built using PAUP v4b10 [60]. The results obtained from nonparametric bootstrapping (using 1000 replicates for NJ and 100 replicates with TBR heuristic searches for MP) were displayed in 50% majority rule bootstrap consensus trees. For Bayesian inference, a restriction site model was used [61]. For each dye, four independent chains, each of length 50 million sampling every 5000 trees, were run using MrBayes v3.1 [62]. Convergence was assessed using Tracer v1.4.1, and a burn-in of 30 million trees was used.

To assess the degree to which the bootstrap consensus trees for the four individual primer combinations were in agreement with each other, we constructed a filtered supernetwork for each NJ and MP bootstrap consensus tree in SplitsTree 4.10 [56]. Supernetworks provide a means to visualize incongruence between potentially large bifurcating trees. Because they generalize supertree construction to networks, they can also facilitate interpretation of evolutionary histories that are complex or uncertain [63,64]. Filtered supernetworks [63,64] emphasize the commonality of the strongest signal from independent datasets, and allow visualization of the support for species delimitation and evolutionary relationships. Even given the stochastic error that can be associated with AFLP fingerprint profiles, supernetworks provide a framework for assessing whether independent data provide corroborating evidence for species limits and evolutionary relationships.

For tree-building analyses of the combined AFLP data, MP, NJ and Bayesian analyses were performed as above. Non parametric bootstrapping of MP and NJ trees was also used to cluster individuals for the combined AFLP profiles, performed as above.

### Bayesian and PCO-MC analyses of genetic structure of the AFLP dataset

We used the program Structure v2.2.3 [42] and PCO-MC [65] to investigate patterns of genetic structure in New Zealand *Ourisia *to compare with and complement the tree-building analyses regarding species delimitation. Structure is a mixture model-based Bayesian clustering method that groups individuals into *K *populations or species and assigns admixture proportions of each individual to these groups. The most recent version of the program is recommended over the previous version (v2.1) for AFLP data because it has been modified to treat dominant markers explicitly [66]. Although not explicitly developed for addressing questions of species delimitation, Structure has been used successfully in a recent study to delimit recently evolved species where clades were not sufficiently distinct to be recovered by tree-based methods [43]. We ran Structure firstly to determine the number of clusters in the dataset and their composition, and secondly to identify any evidence of admixture in each cluster and interpret it in light of the phylogenetic results.

The combined AFLP dataset of 193 individuals was analyzed in Structure using an admixture model and correlated allele frequencies, without incorporating population information into the analyses. Preliminary analyses determined that stationarity of parameters (alpha, ln likelihood, Fst) was achieved by 25,000 generations (data not shown). Thus, for all Structure analyses, we used a total run length of 100,000 generations, including a burn-in of 25,000 generations, testing *K *= 1 to 16 with three separate runs at each *K*. Individuals were assigned to 16 *a priori *"populations" (morphologically-defined species and subspecies groupings following [33]) that were not taken into consideration in the analyses, but were nevertheless used in resulting figures to improve visualization of the results. We plotted the mean -ln likelihood of the data vs. *K*, and Δ*K *vs. *K *for all runs [35], and examined all groupings at each *K*.

We also analyzed the combined *Ourisia *AFLP dataset using PCO-MC, a recently published method [65] which couples principal coordinate analysis (PCO) with a clustering procedure to determine significant population structure. This method offers an objective way to determine whether clusters found in the PCO are significant, and it simultaneously takes into consideration many or all of the axes that explain the variation in a dataset (as opposed to only the first three that can be easily visualised [65]). The PCO-MC analysis was carried out on the combined *Ourisia *AFLP dataset following [65] and  by performing a PCO analysis in NTSYS 2.11× (Exeter Software) using Jaccard distances and DCENTER, EIGEN, and MOD3D modules, and then performing the cluster analysis using the MODECLUS procedure in SAS 9.1 (SAS Institute).

## Authors' contributions

HMM conceived of the study, carried out the field work, generated and scored the AFLP data, performed some data analysis, and drafted the manuscript. DB did programming and data analysis. PJL assisted with data analysis and interpretation, and helped write and revise the manuscript. All authors read and approved the final manuscript.

## Supplementary Material

Additional file 1 Comparison of bootstrap values for monophyletic species and subspecies of New Zealand species of *Ourisia *and certain species relationships for different phylogenetic analyses of the individual AFLP primer datasets. Click here for file

Additional file 2 Bayesian 50% majority rule tree of New Zealand species of *Ourisia*, rooted with one of the Tasmanian individuals of *O. integrifolia *and showing posterior probability (PP) values > 50 for main clades. An asterisk (*) means 100 PP. See Additional file 4 for name abbreviations and voucher information, and see Fig. 3 for the same tree, unrooted. Click here for file

Additional file 3 Proportion of membership of each pre-defined population of New Zealand and Australian *Ourisia *in each of the clusters of the Structure analyses. A. K = 2 clusters, B. K = 3 clusters, C. K = 12 clusters. The highest proportion for each population is shown in **bold underline**, whereas populations with > 0.200 proportion in a second cluster is in ***bold italics***. Click here for file

Additional file 4 Voucher information for all individuals of *Ourisia *used in this study. Taxonomy follows Meudt [33]. Click here for file
