# Supplementary material for: Species delimitation and phylogeny of a New Zealand plant species radiation
Source: BMC Evol Biol. 2009 May 20;9:111. doi: 10.1186/1471-2148-9-111 (PMC2700801; doi:10.1186/1471-2148-9-111)

Meudt et al. *BMC Evolutionary Biology* (2009)

Additional File 2.

Bayesian 50% majority rule tree of New Zealand species of *Ourisia*, rooted with one of the Tasmanian individuals of *O. integrifolia* and showing posterior probability (PP) values >50 for main clades. An asterisk (*) means 100 PP. See Additional File 4 for name abbreviations and voucher information, and see Fig. 3 for the same tree, unrooted.

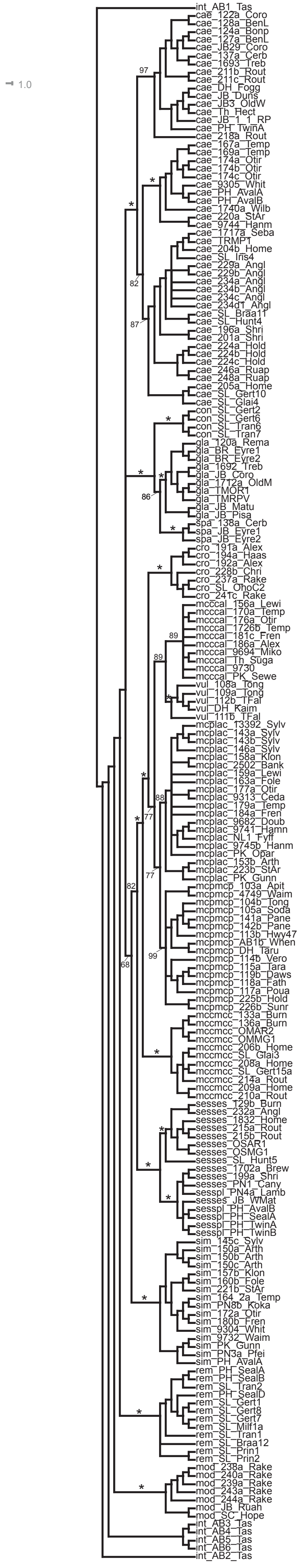

Supplement: Additional file 2 — Bayesian 50% majority rule tree of New Zealand species of Ourisia, rooted with one of the Tasmanian individuals of O. integrifolia and showing posterior probability (PP) values > 50 for main clades. An asterisk (*) means 100 PP. See Additional file 4 for name abbreviations and voucher information, and see Fig. 3 for the same tree, unrooted. [file 1471-2148-9-111-S2.pdf]
